# Iso-Partricin, an Aromatic Analogue of Amphotericin B: How Shining Light on Old Drugs Might Help Create New Ones

**DOI:** 10.3390/antibiotics10091102

**Published:** 2021-09-13

**Authors:** Paweł Szczeblewski, Justyna Górska, Witold Andrałojć, Patryk Janke, Karolina Wąsik, Tomasz Laskowski

**Affiliations:** 1 Department of Pharmaceutical Technology and Biochemistry and BioTechMed Centre, Faculty of Chemistry, Gdańsk University of Technology, Gabriela Narutowicza Str. 11/12, 80-233 Gdańsk, Poland; pawel.szczeblewski@pg.edu.pl (P.S.); just.go.pg@gmail.com (J.G.); patrykjanke@gmail.com (P.J.); karolina.wasik94@gmail.com (K.W.); 2 Institute of Bioorganic Chemistry, Polish Academy of Sciences, Zygmunta Noskowskiego Str. 12/14, 61-704 Poznań, Poland; wandralojc@ibch.poznan.pl

**Keywords:** partricin, aureofacin, polyene macrolides, photoisomerization, selective toxicity, structural studies, NMR

## Abstract

Partricin is a heptaene macrolide antibiotic complex that exhibits exceptional antifungal activity, yet poor selective toxicity, in the pathogen/host system. It consists of two compounds, namely partricin A and B, and both of these molecules incorporate two *cis*-type bonds within their heptaenic chromophores: 28*Z* and 30*Z*. In this contribution, we have proven that partricins are susceptible to a chromophore-straightening photoisomerization process. The occurring 28*Z*→28*E* and 30*Z*→30*E* switches are irreversible in given conditions, and they are the only structural changes observed during the experiment. The obtained *all-trans* partricin’s derivatives, namely iso-partricins A and B, exhibit very promising features, potentially resulting in the improvement of their selective toxicity.

## 1. Introduction

Among several groups of clinically used antifungal antibiotics, polyene macrolides should be regarded as the most versatile and promising family of fungicidal agents. They exhibit most of the features of a mythical “ideal drug”, with two major disadvantages depriving them of their rightful place on top of the antifungal pantheon: (1) poor bioavailability, resulting from poor solubility in water; and (2) relatively poor pathogen/host selective toxicity [1]. Although the former problem may be easily overcome by a proper formulation of a drug, the latter is directly related to the molecular mode of action of clinically used polyene macrolides; thus, they require rational modifications to their chemical structure.

The highest antifungal activity is attributed to the polyene macrolides belonging to the heptaenic group. This family may be divided into two subgroups: (1) non-aromatic *all-trans* heptaenes, with amphotericin B (AmB) being “the big star” and still considered to be a golden standard in treatment of systemic fungal infections, and (2) aromatic heptaenes (AHs), incorporating two *cis*-type double bonds in their heptaenic chromophores and an extra structural feature: an alkyl-aromatic side chain, attached to the major macrolactone ring [2,3]. Members of the latter subgroup exhibit up to two orders of magnitude higher antifungal activity than AmB [4], yet none of them is currently recommended for clinical use in treatment of internal mycoses, mainly due to their especially poor selective toxicity and severe side effects.

**Figure 1:**
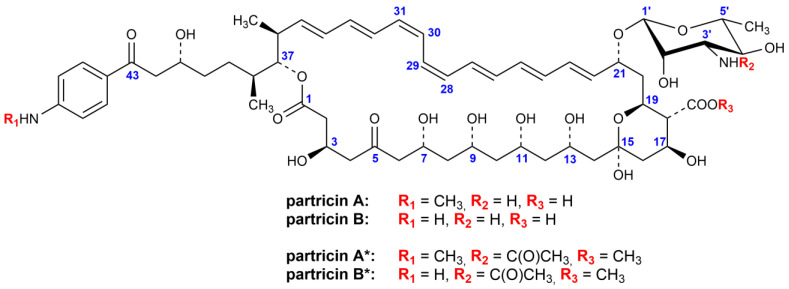
Structure of partricins A and B, along with their methyl esters of N-acetyl derivatives.

AHs are often misrepresented in the literature, as the *all-trans* compounds, yet none of the Ahs—found in nature to this date, that is—exhibits such a geometry of the chromophore. The most widely known representative of AHs, the candicidin complex (syn. levorin, ascosin, produced by *Streptomyces griseus*), consists of three major components and each of them incorporates two *cis*-type bonds, i.e., 26*Z* and 28*Z* [5]. The other member of the AH family, partricin complex (syn. aureofacin, produced by *Streptomyces aureofaciens*) involves two compounds, differentiated solely by one methyl group at the end of the side chain [6,7]. Both of these molecules also incorporate two *cis*-type bonds, yet in different positions, i.e., 28*Z* and 30*Z* (Figure 1) [8,9,10]. Members of the partricin complex, partricin A (syn. gedamycin) and partricin B (syn. vacidin) contain more oxygen functions in C1-C15 fragment than the candicidins [5], which probably translates to slightly higher antifungal activity due to enhanced rigidity of partricin’s macrolactone ring.

Although partricin is not currently used as a weapon against systemic mycoses, methyl ester of partricin, aka mepartricin (Ipertrofan and Tricandil), was proven to be useful in treatment of benign prostatic hyperplasia and chronic nonbacterial prostatitis/pelvic pain syndrome [11,12,13,14]. Similar to all members of the heptaenic family, mepartricin effectively binds to steroids, and this feature is considered to be the foundation of the therapeutic effect [15].

Previously, we have shown that the main candicidin, candicidin D [16], while exposed to UV–VIS radiation, undergoes photochemical isomerization reaction, which results in straightening of the heptaenic chromophore to the AmB-type (*all-trans*) geometry [17]. This process is irreversible in the given conditions and it is the only structural change occurring during the experiment, assuming that the radiation dose and the time of exposure are fairly optimized. Since partricins exhibit different geometry of the heptaenic chromophore [8,10], in this study, we have settled whether their exposure to UV–VIS radiation would produce similar results to the ones observed for candicidin D. Straightening of the heptaenic chromophore to the *all-trans* geometry should be regarded as the first step in a long journey, leading to significant improvement of AHs’ selective toxicity and, thus, creation of a better drug than the current standard, amphotericin B.

## 2. Results

### 2.1. Shedding A New Light to Old Matters

As we stated before [5], there are many inconsistencies in the literature regarding data on aromatic heptaene macrolide antibiotic complexes. Previously, we have solved the candicidin case [17]. Going on with our mission to put these matters in order, we have also confirmed that the names “partricin” and “aureofacin” should be used as synonyms, since they refer to the same antibiotic complex [18]. As we postulated earlier, some of the previous confusions might have risen from the photochemical isomerization phenomenon of the partricin complex, which we decided to examine in this contribution.

**Figure 2:**
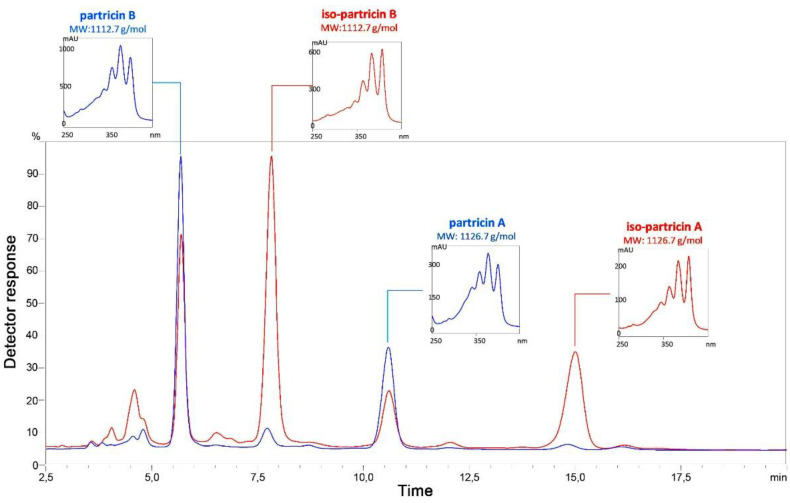
Superimposed HPLC–DAD–ESIMS chromatograms of partricin complex. Red line: sample dissolved and stored at room temperature for 24 h, with an unlimited access to daylight. Blue line: sample dissolved and stored at room temperature in darkness for 24 h (control).

Similarly to the procedure applied in case of the candicidin complex, a simple experiment was conducted [17]. Two identical solutions of partricin complex were prepared: one stored in darkness for 24 h (control) and the second one, simultaneously stored and unguarded from direct daylight. As can be seen in Figure 2, observed number of major components of the partricin complex, illuminated with daylight (red line), has doubled in comparison to the control sample (blue line). Partricins A and B were partially transformed into their isomers, exhibiting the very same molecular masses, yet responding with significantly altered UV–VIS spectra. Since the heptaenic chromophore of polyene macrolides is by far the major contributor to their electronic spectra, it was obvious that structural changes—at least those registered by photonic absorption—must have occurred in this region.

UV–VIS spectra of the native partricins incorporate three absorption maxima at λ1 = 360 nm, λ2 = 378 nm and λ3 = 401 nm, with the one at the medium (λ2) being of highest intensity (Figure 2). Such an image is generally attributed to a heptaenic system that contains several *Z* double bonds within [19]. While the positions and number of the *Z* bonds might not be directly extracted from the antibiotics’ electronic spectra, previous studies on partricin A and partricin B have proven that those molecules contain two *cis*-type double bonds at positions 28 and 30 (Figure 1) [8,10].

The absorption maxima observed for iso-partricins A and B were subjects to bathochromic shifts, manifesting at λ1* = 364 nm, λ2* = 384 nm and λ3* = 407 nm. Moreover, the relative intensities of λ2* and λ3* were inversed in relation to the native compounds (Figure 2). This shape of an electronic spectrum has been previously demonstrated to indicate to the *all-trans* type of heptaenic chromophore, identical to the one of amphotericin B [3]. However, this testimony could not be accepted as a direct structural proof and, therefore, required further verification. Additionally, the remaining structural regions of the iso-molecules must have also been tested for other potential light-induced constitutional alterations. Thus, ^1^H NMR was selected as a major tool for the following structural studies.

### 2.2. NMR Studies on Iso-Partricin A and B

The native partricin complex was derivatized into its N-acetyl methyl ester via series of simple reactions. This procedure, developed at our department, is a well-established approach for the elucidation of constitution and/or stereochemistry of polyene macrolides, using NMR techniques [5,16,20,21,22], and, as an option, could be followed in order to facilitate purification of studied antibiotic molecules and enhance their solubility in standard NMR solvents. Later, the resulting methyl ester of N-acetylpartricin (referred to as partricin*) was exposed to moderate UV–VIS radiation of λ = 365 nm. The chosen reaction wavelength was identical to the one that produced the best results for candicidin D and was not optimized in this study [17]. The progress of a photochemical reaction was traced by using continuous UV–VIS spectra. When further irradiation caused no visible changes in the electronic spectra, methyl ester of N-acetyl-iso-partricin A (referred to as iso-partricin A*) and methyl ester of N-acetyl-iso-partricin B (aka iso-partricin B*) were isolated and purified by using HPLC.

Iso-partricin A* and iso-partricin B* molecules were subjects to a standard set of 2D NMR experiments, consisting of DQF-COSY, TOCSY, HSQC, HMBC and ROESY spectra (please consult Figures S1–S12) [5,16,20,21,22,23,24,25,26]. DQF-COSY, TOCSY and HSQC spectra were used to trace proton–proton and proton–carbon connectivities within isolated protonic spin systems, while HMBC and ROESY experiments enabled gluing all the pieces together, due to long ranged heteronuclear couplings and structurally conclusive dipolar couplings between protons. Finally, DQF-COSY and ROESY spectra allowed the definition of the stereostructure of the studied compounds, including absolute configurations of almost all stereogenic centers within the molecules and—most importantly—the geometries of iso-partricin’s A* and iso-partricin’s B* chromophores.

**Figure 3:**
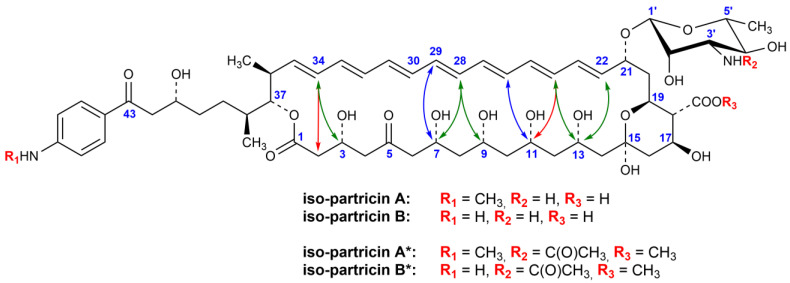
Structure of iso-partricins A and B, along with their methyl esters of N-acetyl derivatives. Dipolar couplings between the protons of the chromophores and the polyol chains are depicted as bidirectional arrows. Colors: blue—ROEs observed only for the native partricins [8,10]; green—ROEs observed for both native and isomeric partricins; red—ROEs observed only for the *all-trans* iso-partricins.

Detailed NMR studies revealed that, in contrary to the NMR data on the partricin A and partricin B methoxycarbonylmethylamide derivatives [8,9,10], all the ^3^J_H,H_ coupling constants within the double bonds were no lower than 15.1 Hz and no higher than 15.6 Hz, thus determining the *E* geometry of the entire chromophore systems. The chemical shifts of all the olefinic carbons (C22–C35) ranged between 130 and 137 ppm, which strongly suggested the *E* geometries of all the double bonds within the chromophores, since no shielding γ-effects were observed for the C27/C30 and C29/C32 pairs [9,16,22]. Moreover, rich sets of ROEs between the protons of the C2–C13 and C22–C35 fragments were recorded (Figure 3), as well as two uninterrupted ROE pathways, incorporating even- and odd-numbered olefinic protons (see Appendix A section, Table A1). The stereostructural requirements for all those dipolar couplings might be met only in case of straightening of the heptaenic chromophores to the *all-trans* geometries. Additionally, some of the vicinal proton–proton coupling constants within the C2–C9 fragments have changed in comparison to the *cis–trans* derivatives [9,10], which should be attributed to the alteration of the flexibility of the macrolactone ring systems, resulting from changes in the geometry of the chromophores.

No other constitutional and/or stereochemical changes in iso-partricin A* and iso-partricin B* were found in comparison to the native compounds. Thus, we have proven that 28*Z*→28*E* and 30*Z*→30*E* switches are the only chemical changes of partricins’ structures, occurring as a result of moderate UV–VIS irradiation.

More detailed information on the ^1^ H and ^13^ C resonances of iso-partricin A* and iso-partricin B* is given in Table A1.

## 3. Discussion

The work presented in this manuscript, along with the previously conducted studies on candicidin D and its *all-trans* isomer [17], have proven that the native aromatic heptaene macrolide antifungal antibiotics are in fact susceptible to a chromophore-straightening photoisomerization process, regardless of the positions of the *Z* double bonds within their original chromophores. The resulting transformations are irreversible in the given experimental conditions, yet it must be noted that the yield of the whole process is below 100%, since the welcomed structural changes are in fact competed by the antibiotics’ degradation. The latter fact presumably creates a space for further optimization of the production of the AHs’ isoforms.

The *all-trans* aromatic heptaenes (especially the iso-partricins, exhibiting high resemblance of their polyol chains to the one of AmB) might be, therefore, considered as aromatic analogues of amphotericin B—the only polyene macrolide antibiotic used clinically in treatment of systemic fungal infections. Our preliminary results on in vitro biological activity of iso-candicidin D, iso-partricin A and iso-partricin B have strongly suggested that fungicidal activity of the *all-trans* isoforms remains comparable to the native molecules, thus still exceeding the one of AmB by almost two orders of magnitude. Meanwhile, the hemolytic activity of iso-AHs has been substantially reduced in comparison to the native *cis–trans* forms. The calculated selective toxicity indexes (STIs), which relate to the EH_50_ to MIC ratio, were equal to > 20 and 19.28 for iso-partricin A and iso-partricin B, respectively. Initial assessments have therefore demonstrated that both iso-partricins were more selective than AmB (STI = 13.84), mainly due to the lowered hemolytic activity. For more details on biological activity of iso-partricins, along with computational studies on their interactions with membrane sterols, please consult [27].

In the end, the in vitro selective toxicity index of AHs seems to benefit a lot from the straightening of their heptaenic chromophores, which encourages us to have high hopes, regarding following studies on these compounds and their further development.

## 4. Materials and Methods

### 4.1. Partricin Complex

The crude partricin complex was obtained by extraction with n-butanol from fermentative broth of *Streptomyces aureofaciens NRRL 3878* in the Department of Pharmaceutical Technology and Biochemistry, Gdańsk University of Technology (Gdańsk, Poland). The volume of resulting solution was reduced by evaporation under reduced pressure and centrifuged. The precipitate was washed several times with acetone and dry ethyl ether and dried under reduced pressure. The crude antibiotic complexes were then purified by using the procedure described in patent no. 83,710 [28].

### 4.2. Synthesis of Methyl Ester of 3′-N-Acetylpartricin Complex (Partricin*)

Derivatization of the purified partricin complex was performed with the general procedure previously elaborated in our laboratory and described in References [5,21,22].

### 4.3. Photochemical Cis−Trans Isomerization of Partricin*

The partricin* complex was dissolved in a 95% methanol/5% water solvent system to a concentration of 1 mg/mL. While gently stirring, one liter of this solution (~2 cm depth) was then irradiated with two long-wavelength UV lamps (λ = 365 nm, 8 W) for ca. 1 h. The reaction vessel was placed in the dark, at room temperature. The progress of the photochemical isomerization reaction was monitored by using UV−VIS spectroscopy and RP-HPLC analysis. The RP-HPLC analysis conditions were as follows: column, Luna 100 C18 (2) (150 × 4.6 mm, 5 µm); mobile phase composition, 38% acetonitrile/62% ammonium acetate buffer (5.5 mmol, pH = 4.5), *v*/*v*; flow rate, 1 mL/min; detection at 378 nm; room temperature.

### 4.4. Isolation of the Methyl Esters of 3′-N-Acetyl-Iso-Partricin A and B (Iso-Partricin A* and Iso-Partricin B*)

The isolation of the iso-partricin A* and iso-partricin B* from the partricin* complex was performed by means of semi-preparative HPLC on a Merck–Hitachi apparatus L-6200A, equipped with Merck–Hitachi L-4250 UV–VIS detector. The separation conditions were as follows: column LiChrosorb Si60 (250 mm × 10 mm, 7 μm), mobile phase composition: chloroform/methanol/water (5:0.4:0.035, *v*/*v*/*v*); flow rate 6.25 mL/min; detection at 407 nm, room temperature. A sample of 10 mg/mL (dissolved in the mobile phase) in a volume of 0.625 mL was injected. The retention time was 18 min and 29 min for iso-partricin A* and iso-partricin B*, respectively. The semi-preparative HPLC separation was performed several times, yielding 7 mg of the iso-partricin A* and 8 mg of the iso-partricin B*.

### 4.5. NMR Experiments

The NMR spectra were recorded with a Bruker Avance III HD 700 MHz spectrometer equipped with QCI CryoProbe in solvent system pyridine-*d_5_*-methanol-*d_4_*, 9:1 (*v*/*v*) at 25 °C with a sample concentration of 15 mg/mL. Chemical shifts were reported in δ (ppm) units, using ^1^H residual resonance from pyridine-*d_5_* (7.19 ppm) as internal standard. The 1D ^1^ H NMR spectra were collected with digital resolution of 0.5 Hz. The ^1^ H 90° pulse length was 7.0 µs.

Two-dimensional ^1^H spectra were measured in the phase-sensitive mode with a spectral width of 7704 Hz.

The DQF-COSY spectra were acquired in a 4096 × 512 matrix with 32 accumulations per increment and were processed in a 4K × 2K matrix.

The TOCSY spectra were acquired with a mix time of 60 ms in a 2048 × 512 matrix with 32 accumulations per increment in a 2K × 1K matrix.

The ROESY spectra were acquired with a mix time of 300 ms in a 2048 × 512 matrix with 64 accumulations per increment in a 2K × 1K matrix.

HSQC and HMBC experiments were performed with pulse field gradients.

The edited HSQC spectra were acquired in the phase-sensitive mode with ^1^J_(CH)_ set to 140 Hz. The spectral windows for ^1^H and ^13^ C axes were 7716 and 29,177 Hz, respectively. The data were collected with 64 accumulations per increment in a 2048 × 256 matrix and processed in a 2K × 1K matrix.

The HMBC spectra were acquired in absolute value mode with ^n^J_(CH)_ set to 9 Hz. The spectral windows for ^1^H and ^13^ C axes were 7716 and 40,515 Hz, respectively. The data were collected with 192 accumulations per increment in a 2048 × 256 matrix and processed in a 2K × 1K matrix.

## Data Availability

The data presented in this study, including high resolution NMR spectra, are available on request from the corresponding author.

## Supplementary Materials

The following are available online at https://www.mdpi.com/article/10.3390/antibiotics10091102/s1. Figure S1: The ^1^ H NMR spectrum of iso-partricin A. Figure S2: The ^1^ H NMR spectrum of iso-partricin B. Figure S3: The DQF-COSY spectrum of iso-partricin A. Figure S4: The DQF-COSY spectrum of iso-partricin B. Figure S5: The TOCSY spectrum of iso-partricin A. Figure S6: The TOCSY spectrum of iso-partricin B. Figure S7: The ROESY spectrum of iso-partricin A. Figure S8: The ROESY spectrum of iso-partricin B. Figure S9: Edited ^1^ H-^13^ C HSQC spectrum of iso-partricin A. Figure S10: The ^1^ H-^13^ C HMBC spectrum of iso-partricin A. Figure S11: Edited ^1^H-^13^C HSQC spectrum of iso-partricin B. Figure S12: The ^1^H-^13^C HMBC spectrum of iso-partricin B, Figure S13: HPLC-DAD-ESIMS chromatogram of isolated iso-partricin A. Figure S14: HPLC-DAD-ESIMS chromatogram of isolated iso-partricin B.

## Appendix A

**Table A1:** ^1^H and ^13^C NMR spectroscopic data for iso-partricin A* and B* (700 MHz, C_6_D_5_N/CD_3_OD (9:1, *v*/*v*)). In every column, the letter A stands for spectroscopic data for iso-partricin A*, and the letter B stands for the analogous value for iso-partricin B*, whereas no letter given means that the presented data are identical for both antibiotics.

Iso-Partricin A* and B*					
Position	δ_C_, Type		δ_H_	J_H,H_ (Hz)	ROE Contacts
*Aglycone*					
1	170.85,	C	–	–	–
2ab ^1^	43.48,	CH_2_	2.796	? (3) ^1^	3, 4a, 4b
3	64.18,	CH	4.863	? (2ab) ^1^, 8.8 (4a), 4.3 (4b)	2ab, 4a, 4b, 34, Me38
4a	51.05,	CH_2_	2.849	8.8 (3), 16.8 (4b)	2ab, 3, 4b
4b			2.990	4.3 (3), 16.8 (4b)	2ab, 3, 4a
5	208.54,	C	–	–	–
6a	51.55,	CH_2_	2.632	16.4 (6b), 1.9 (7)	4a, 6b, 7
6b			2.874	16.4 (6a), 10.2 (7)	4b, 6a, 7
7	67.79,	CH	4.591	1.9 (6a), 10.2 (6b), 2.0 (8a), 9.7 (8b)	6a, 6b, 8a, 8b, 9, 28
8a	43.77,	CH_2_	1.529	2.0 (7), 13.8 (8b), 1.9 (9)	6a, 7, 8b, 9, 10a
8b			1.732	9.7 (7), 13.8 (8b), 10.0 (9)	6b, 7, 8a, 10b
9	72.79,	CH	4.269	1.9 (8a), 10.0 (8b), 2.2 (10a), 9.8 (10b)	7, 8a, 10a, 11, 28
10a	44.49,	CH_2_	1.465	2.2 (9), 14.1 (10b), 1.9 (11)	8a, 9, 10b, 11, 12a
10b			1.670	9.8 (9), 14.1 (10a), 10.3 (11)	8b, 10a, 11
11	73.02,	CH	4.283	1.9 (10a), 10.3 (10b), 2.2 (12a), 9.4 (12b)	9, 10a, 10b, 12a, 13, 24
12a	44.31,	CH_2_	1.411	2.2 (11), 13.2 (12b), 1.8 (13)	10a, 11, 12b, 13, 14a
12b			1.685	9.4 (11), 13.2 (12a), 10.1 (13)	12a, 14b
13	69.11,	CH	4.746	1.8 (12a), 10.1 (12b), 2.0 (14a), 9.4 (14b)	11, 12a, 14a, 22, 24
14a	46.71,	CH_2_	1.755	2.0 (13), 14.6 (14b)	12a, 13, 14b
14b			1.953	9.4 (13), 14.6 (14a)	12b, 14a, 16b
15	98.11,	C	–	–	–
16a	45.28,	CH_2_	1.743	12.4 (16b), 10.3 (17)	16b, 18
16b			2.543	12.4 (16a), 4.5 (17)	14b, 16a, 17
17	66.34,	CH	5.006	10.3 (16a), 4.5 (16b), 10.2 (18)	16b, 18, 19
18	58.39,	CH	2.853	10.2 (17), 10.1 (19)	16a, 17, 19, 20a, 21
19	66.21,	CH	5.049	10.1 (18), 10.5 (20a)	17, 18, 20a, 20b, 22, 1′, 2′
20a	37.86,	CH_2_	2.013	10.5 (19), 15.6 (20b)	18, 19, 20b, 21
20b			2.431	15.6 (20a), 9.6 (21)	19, 20a, 21, 1′
21	75.91,	CH	4.924	9.6 (20b), 9.2 (22)	18, 20a, 20b, 22, 1′
22	136.89,	CH	6.403	9.2 (21), 15.3 (23)	13, 19, 21, 24
23	132.93,	CH	6.449	15.3 (22), 11.1 (24)	25
24	133.90,	CH	6.674	11.1 (23), 15.4 (25)	11, 13, 22, 26
25	130.00,	CH	6.380	15.4 (24), 10.9 (26)	23, 27
26	133.89,	CH	6.476	10.9 (25), 15.1 (27)	24, 28
27	132.42,	CH	6.299	15.1(26), 11.0 (28)	27, 29
28	133.90,	CH	6.649	11.0 (27), 15.6 (29)	7, 9, 26, 30
29	133.88,	CH	6.630	15.6 (28), 10.9 (30)	27, 31
30	133.25,	CH	6.496	10.9 (29), 15.4 (31)	28, 32
31	133.91,	CH	6.673	15.4 (30), 11.3 (32)	29, 33
32	133.01,	CH	6.405	11.3 (31), 15.1 (33)	30, 34
33	133.84,	CH	6.624	15.1 (32), 11.0 (34)	31, 35
34	132.49,	CH	6.301	11.0 (33), 15.5 (35)	2ab, 3, 32, 36
35	136.99,	CH	5.598	15.5 (34), 9.3 (36)	33, 36, 37, Me36
36	40.14,	CH	2.542	9.3 (35), 9.2 (37), 6.8 (Me36)	34, 35, 37, 39a, 39b, Me36, Me38
37	78.63,	CH	5.080	9.2 (36), 2.9 (38)	35, 36, 38, 39a, Me36, Me38
38	33.81,	CH	1.927	2.9 (37), ? (39a, 39b) ^2^, 6.7 (Me38)	37, 39a, 39b, 40ab, 41, Me36, Me38
39a	30.84,	CH_2_	A: 1.719 B: 1.714	? (38, 39b) ^2^, ? (40ab) ^1^	36, 37, 38, 39b, 40ab, 41, Me38
39b			A: 1.761 B: 1.757	? (38, 39a) ^2^, ? (40ab) ^1^	36, 37, 38, 39a, 40ab, 41, Me38
40ab ^1^	A: 35.64, B: 35.66,	CH_2_	A: 1.895 B: 1.877	? (39a, 39b, 41) ^1^	38, 39a, 39b, 41, 42a, 42b, Me38
41	A: 68.35, B: 68.28,	CH	A: 4.609 B: 4.592	? (40ab)1, 3.5 (42a), 9.1 (42b)	38, 39a, 39b, 40ab, 42a, 42b, 45/45′
42a	A: 46.11, B: 46.04,	CH_2_	A: 3.233 B: 3.211	3.5 (41), 15.2 (42b)	40ab, 41, 42b, 45/45′
42b			A: 3.435 B: 3.410	9.1 (41), 15.2 (42a)	40ab, 41, 42a, 45/45′
43	A: 197.76, B: 197.67,	C	–	–	–
Me36	16.48,	CH_3_	0.972	6.8 (36)	35, 36, 37, 38
Me38	12.91,	CH_3_	1.040	6.7 (38)	36, 37, 38, 39a, 39b
COOMe	173.91,	C	–	–	–
COOMe	51.66,	CH_3_	3.818	–	2′, 3′
NHMe	A: 29.36, B: –,	CH_3_	A: 2.811 B: –	–	46/46′
*Aromatic moiety*					
45/45′	A: 131.05, B: 131.23,	CH	8.161	8.6 (46/46′)	41, 42a, 42b, 46/46′
46/46′	A: 110.94, B: 110.24,	CH	6.757	8.6 (45/45′)	45/45′, NHMe
C*CO	A: 154.38, B: 154.25,	C	–	–	–
C*NH	A: 126.12, B: 126.64,	C	–	–	–
*Mycosamine moiety*					
1′	98.16,	CH	4.974	1.9 (2′)	2′, 3′, 5′, 19, 20b, 21
2′	70.79,	CH	4.444	1.9 (1′), 3.5 (3′)	1′, 3′, 19, COOMe
3′	56.09,	CH	4.677	3.5 (2′), 9.4 (4′), 4.5 (NHCOMe)	1′, 2′, 5′, COOMe, NHCOMe
4′	74.62,	CH	4.034	9.4 (3′), 9.5 (5′)	6′, NHCOMe
5′	74.69,	CH	3.790	9.5 (4)’, 6.3 (6′)	1′, 3′, 6′
6′	18.41,	CH_3_	1.591	6.3 (5′)	4′, 5′
NHCOMe	22.83,	CH_3_	2.113	–	3′, NHCOMe
NHCOMe	170.90,	C	–	–	–
NHCOMe	–	–	8.850	4.5 (3′)	4′, NHCOMe

^1^ These protons were perfectly superimposed; hence, the values of the coupling constants involving protons 2a, 2b, 40a and 40b could not be measured. ^2^ These coupling constants could not be measured due to severe signal overlaps and higher order effects.
