# Supplementary material for: Iso-Partricin, an Aromatic Analogue of Amphotericin B: How Shining Light on Old Drugs Might Help Create New Ones
Source: Antibiotics (Basel). 2021 Sep 13;10(9):1102. doi: 10.3390/antibiotics10091102 (PMC8470227; doi:10.3390/antibiotics10091102)
Supplement: Supplementary file 1 [file antibiotics-10-01102-s001.zip › antibiotics-1364664-supplementary.pdf]

antibiotics

MDPI

Article

Iso-Partricin, an Aromatic Analogue of Amphotericin B: How Shining Light on Old Drugs Might Help Create New Ones

Paweł Szczeblewski [1], Justyna Górska [1], Witold Andrałojć [2], Patryk Janke [1], Karolina Wąsik [1] and Tomasz Laskowski [1,*]

[1] Department of Pharmaceutical Technology and Biochemistry, Faculty of Chemistry, Gdańsk University of Technology, Gdańsk, Gabriela Narutowicza Str. 11/12, 80-233, Poland

[2] Institute of Bioorganic Chemistry, Polish Academy of Sciences, Poznań, Zygmunta Noskowskiego Str. 12/14, 61-704, Poland

* Correspondence: tomasz.laskowski@pg.edu.pl; Tel.: +48 58 347 20 79

Supplementary Information

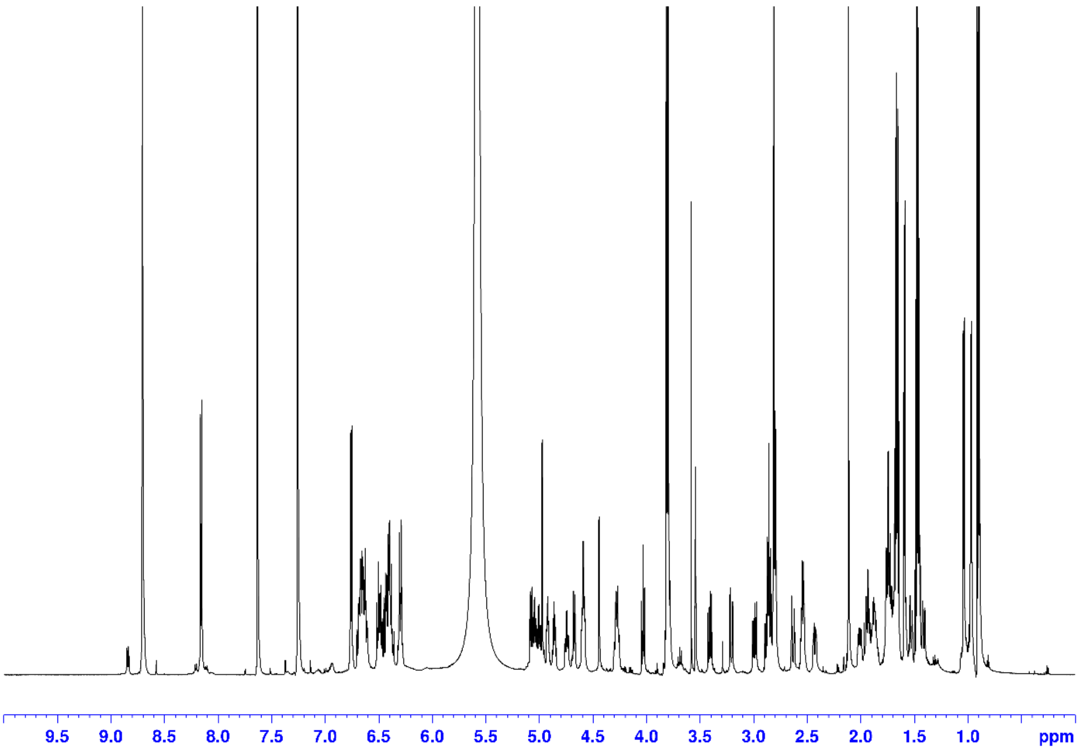

Figure S1. The ^{1}H NMR spectrum of iso-partricin A.

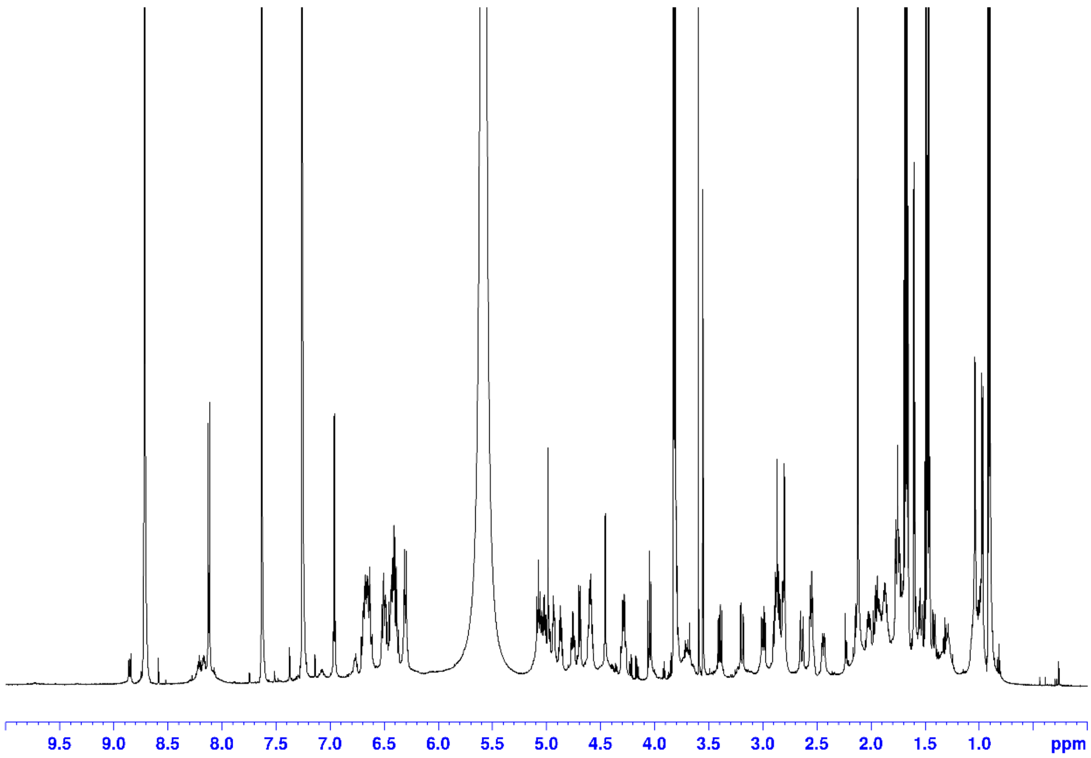

Figure S2. The ^{1}H NMR spectrum of iso-partricin B.

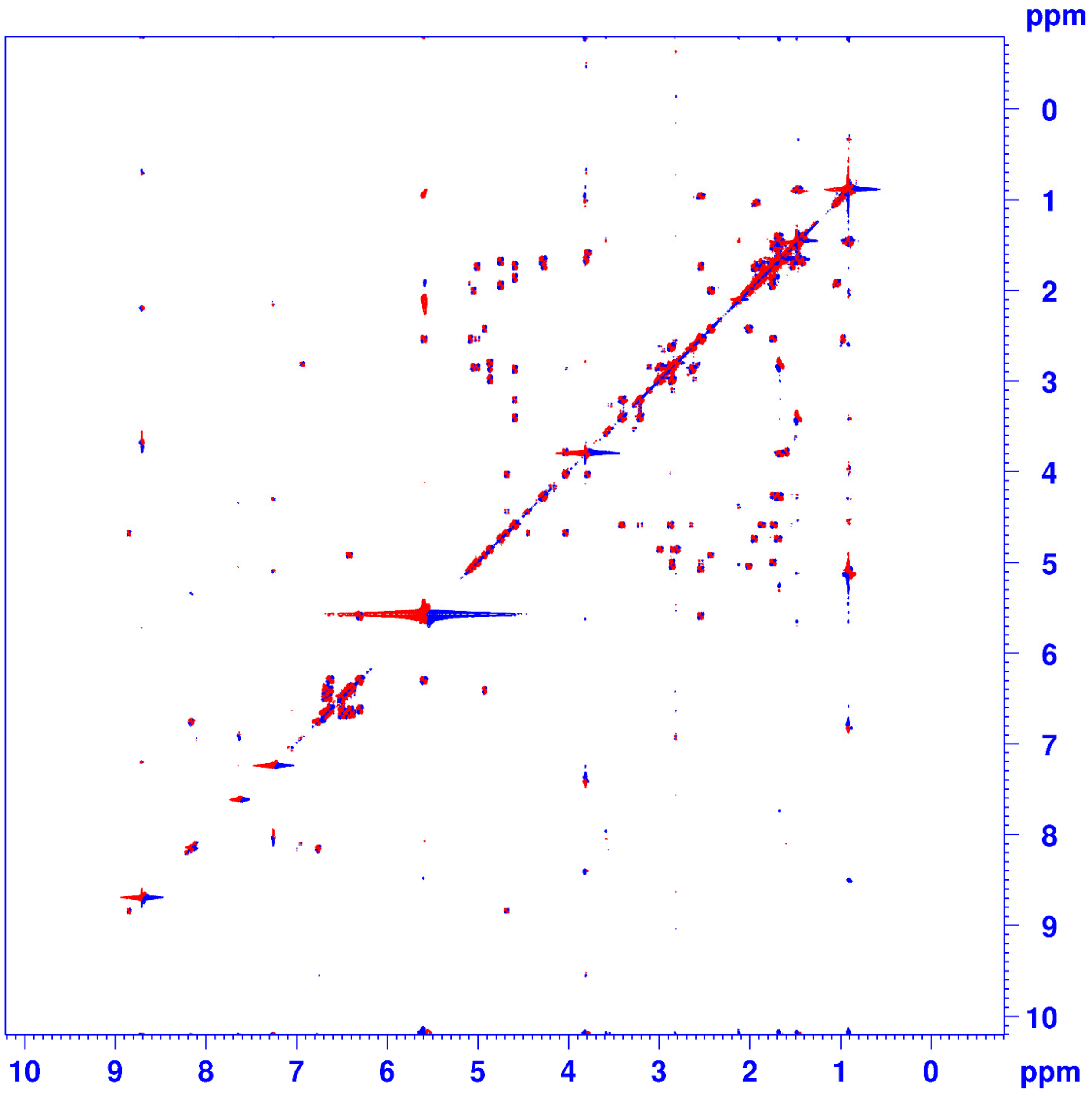

Figure S3. The DQF-COSY spectrum of iso-partricin A.

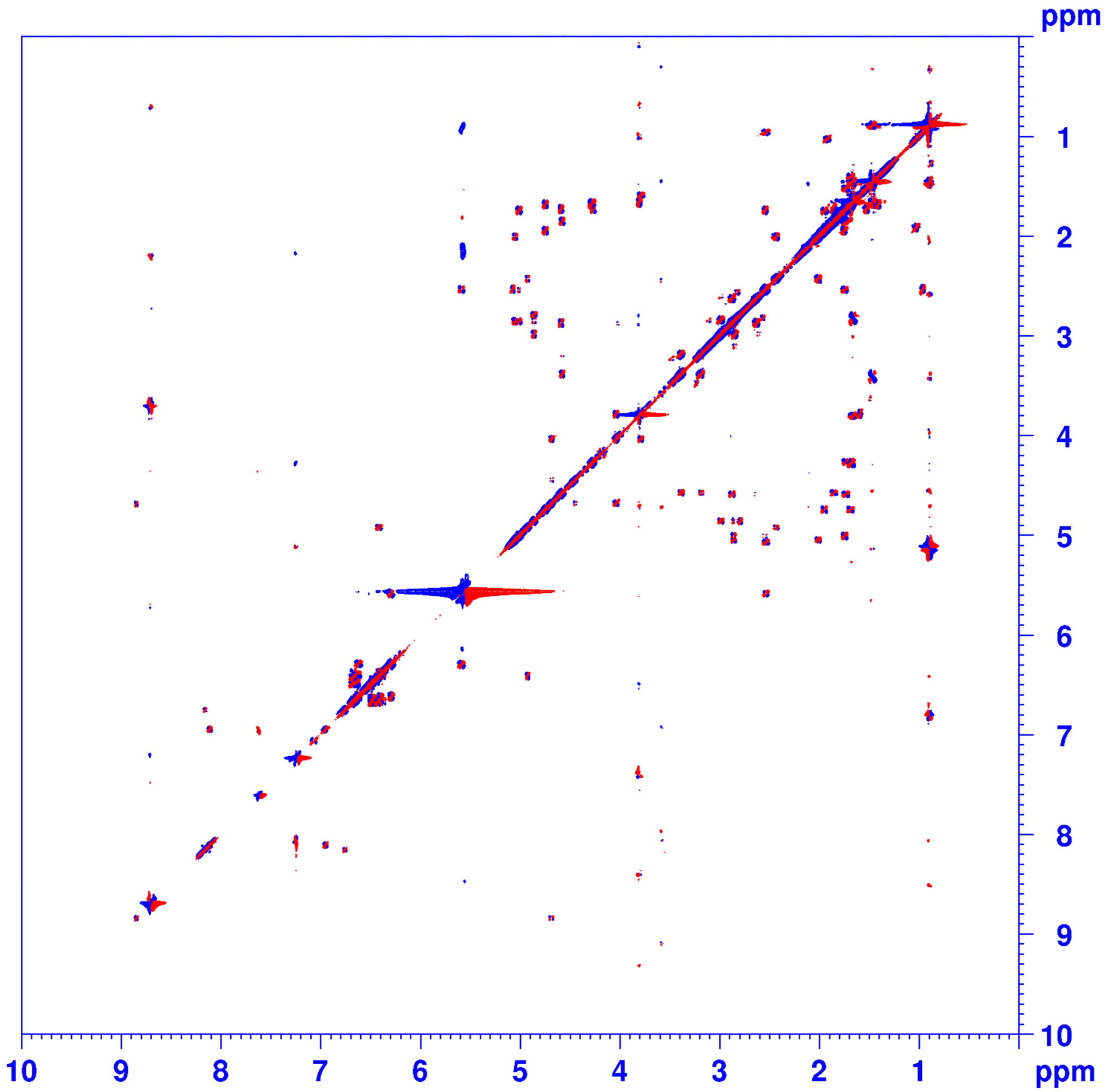

Figure S4. The DQF-COSY spectrum of iso-partricin B.

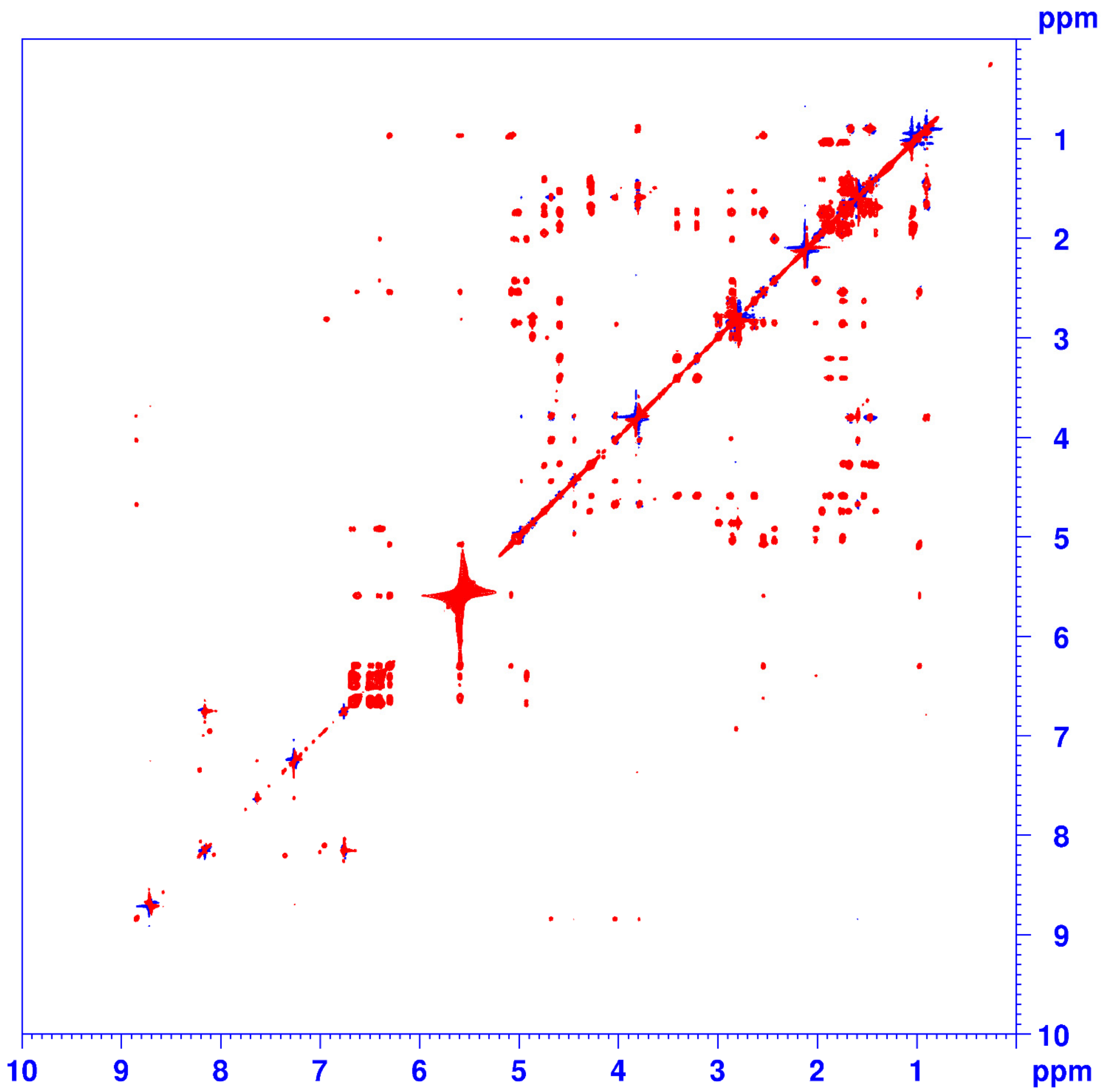

Figure S5. The TOCSY spectrum of iso-partricin A.

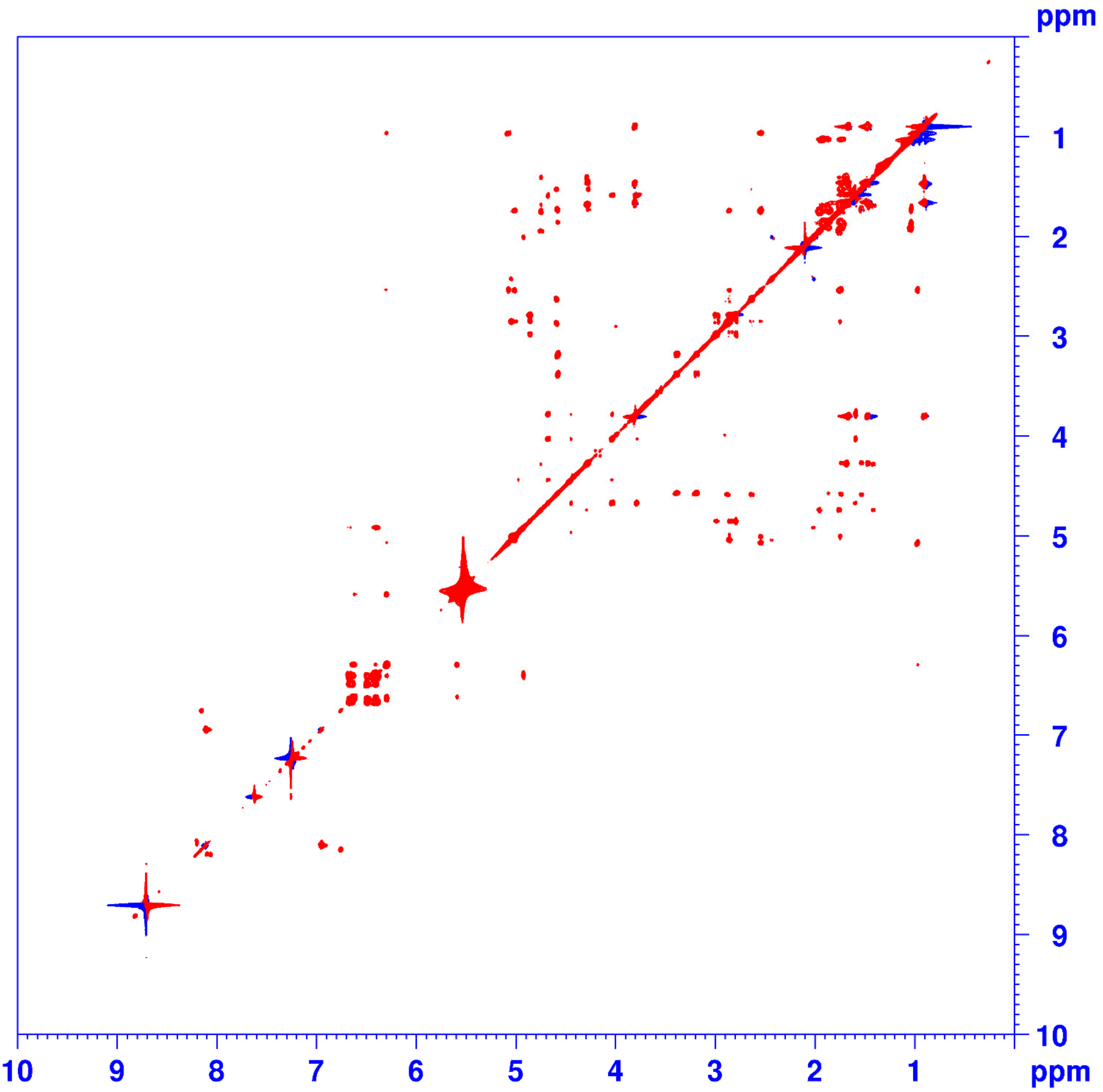

Figure S6. The TOCSY spectrum of iso-partricin B.

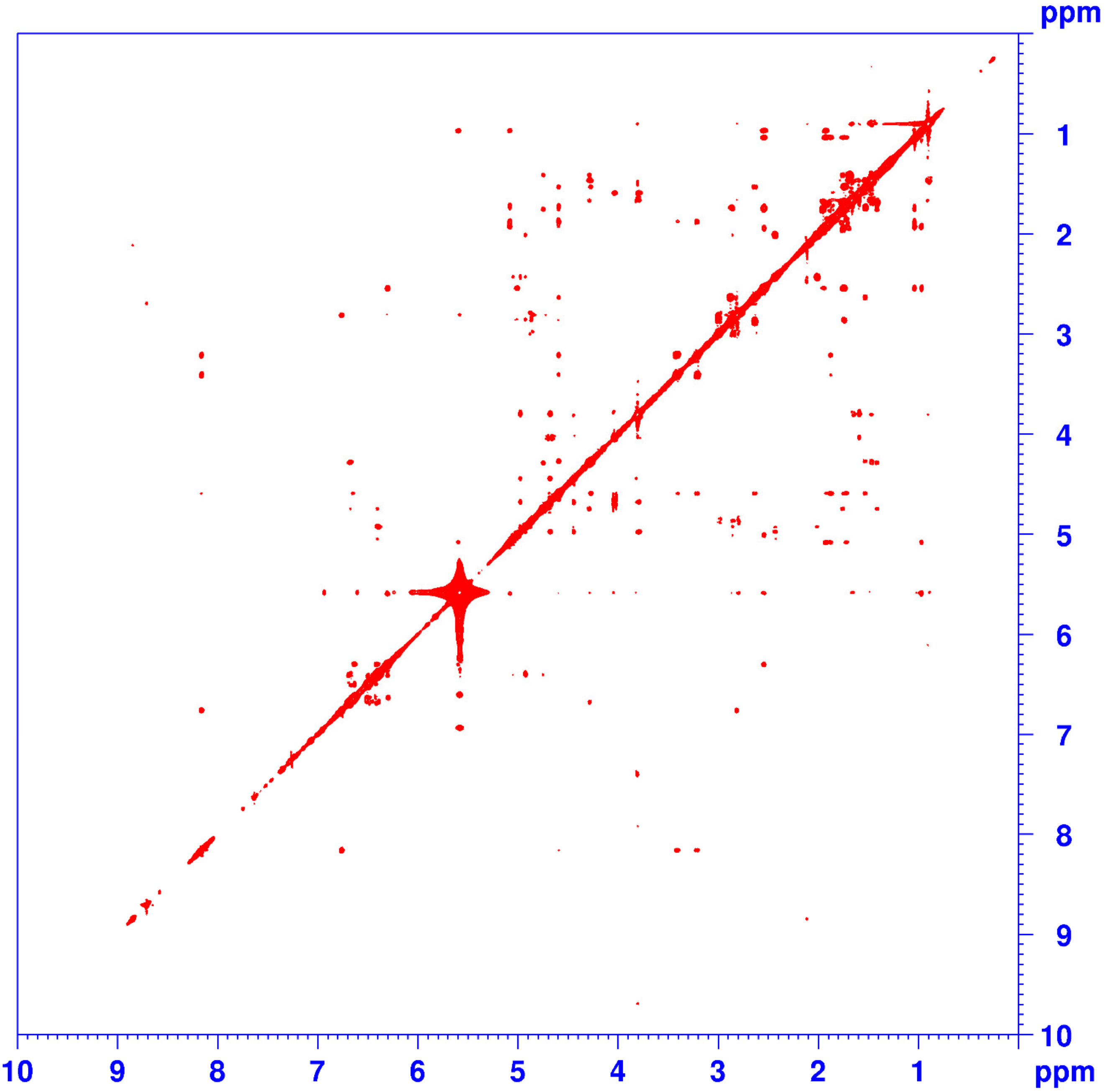

Figure S7. The ROESY spectrum of iso-partricin A.

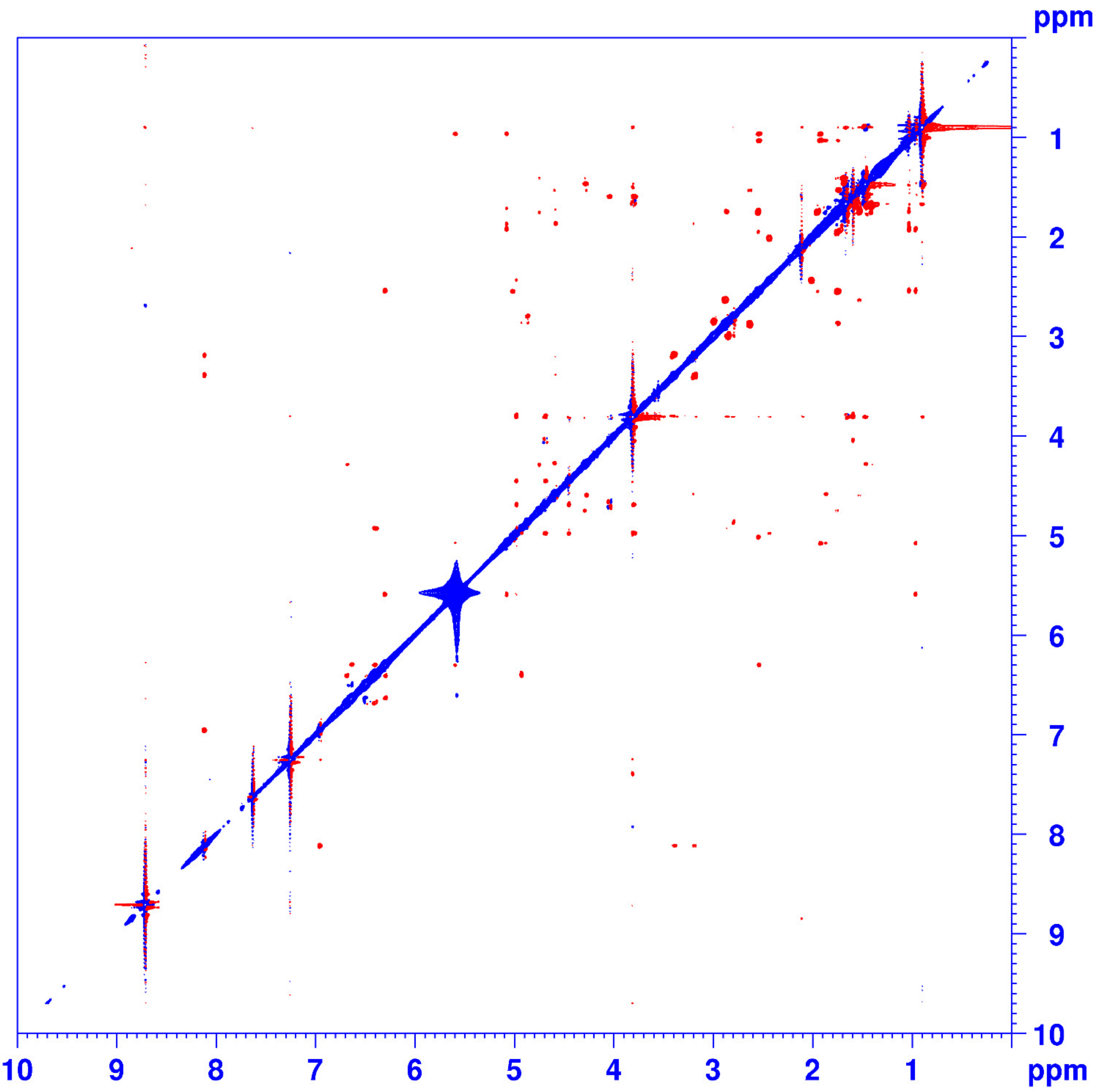

Figure S8. The ROESY spectrum of iso-partricin B.

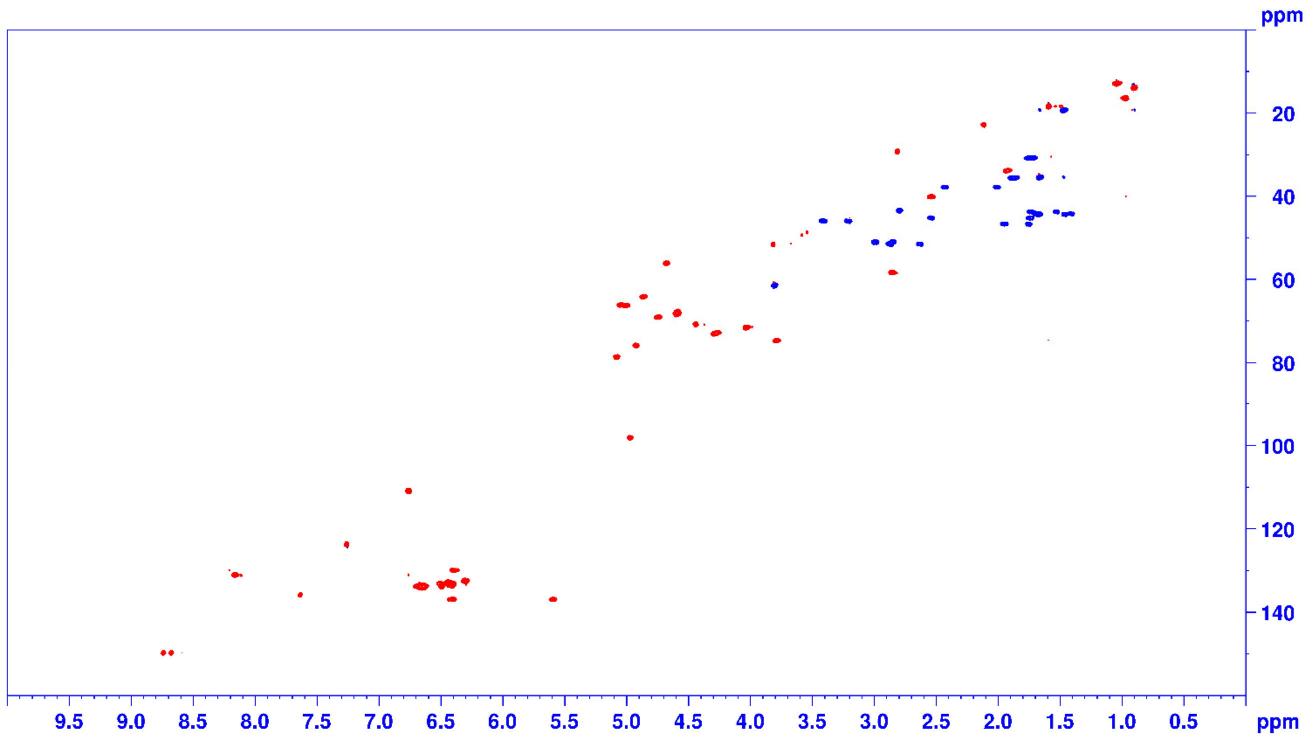

Figure S9. Edited ^{1}H-^{13}C HSQC spectrum of iso-partricin A.

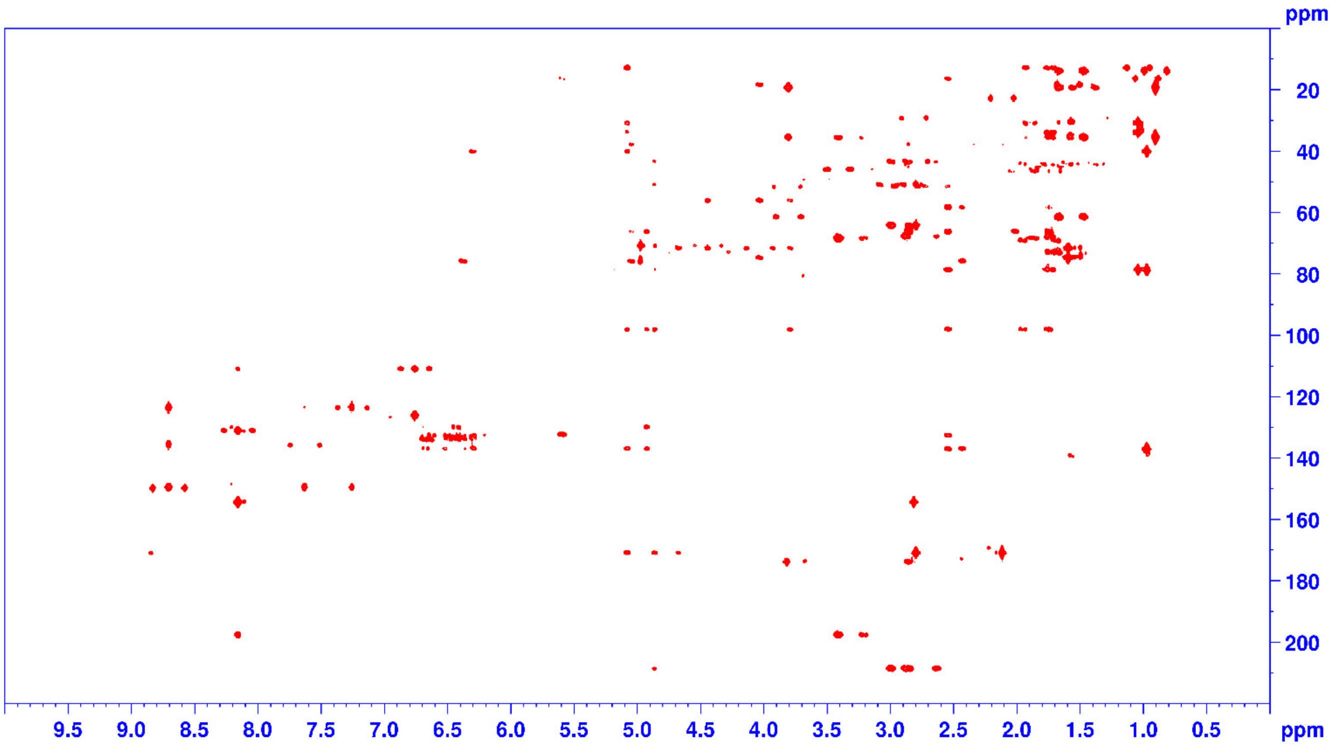

Figure S10. The ^{1}H-^{13}C HMBC spectrum of iso-partricin A.

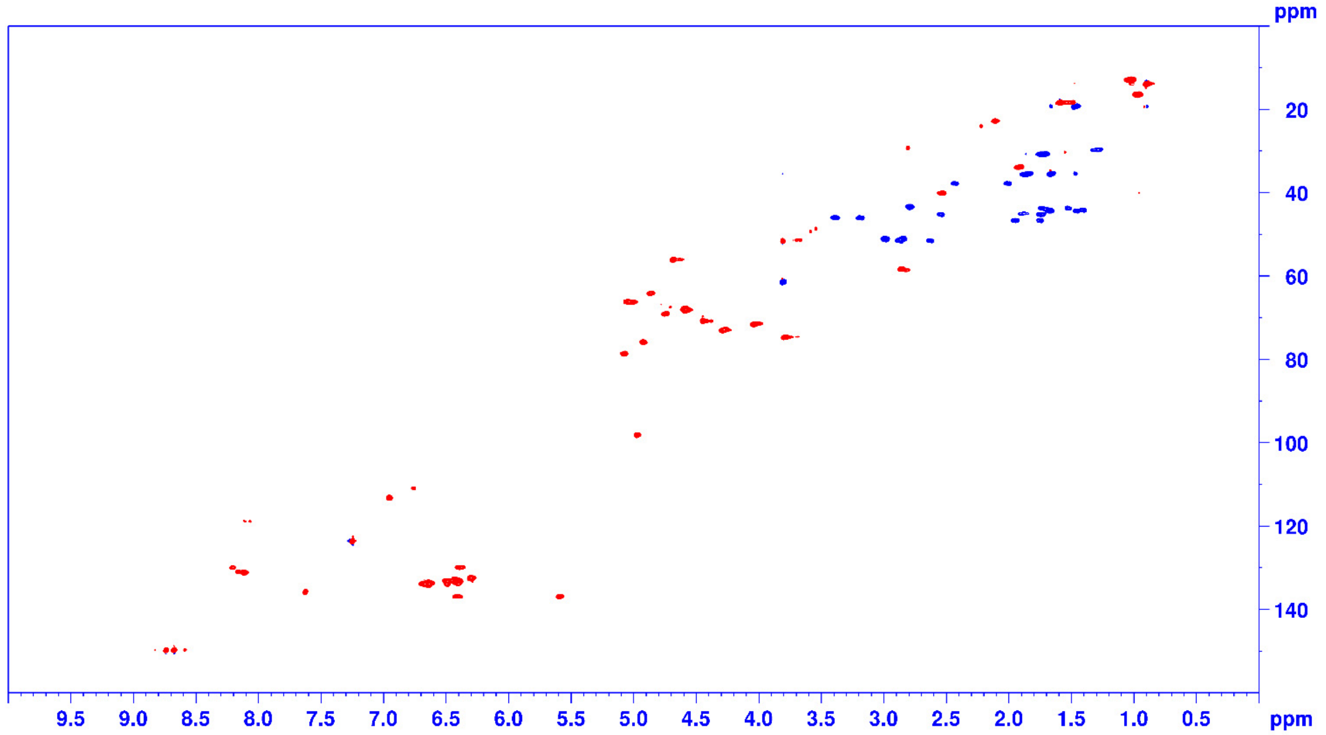

Figure S11. Edited ^{1}H-^{13}C HSQC spectrum of iso-partricin B.

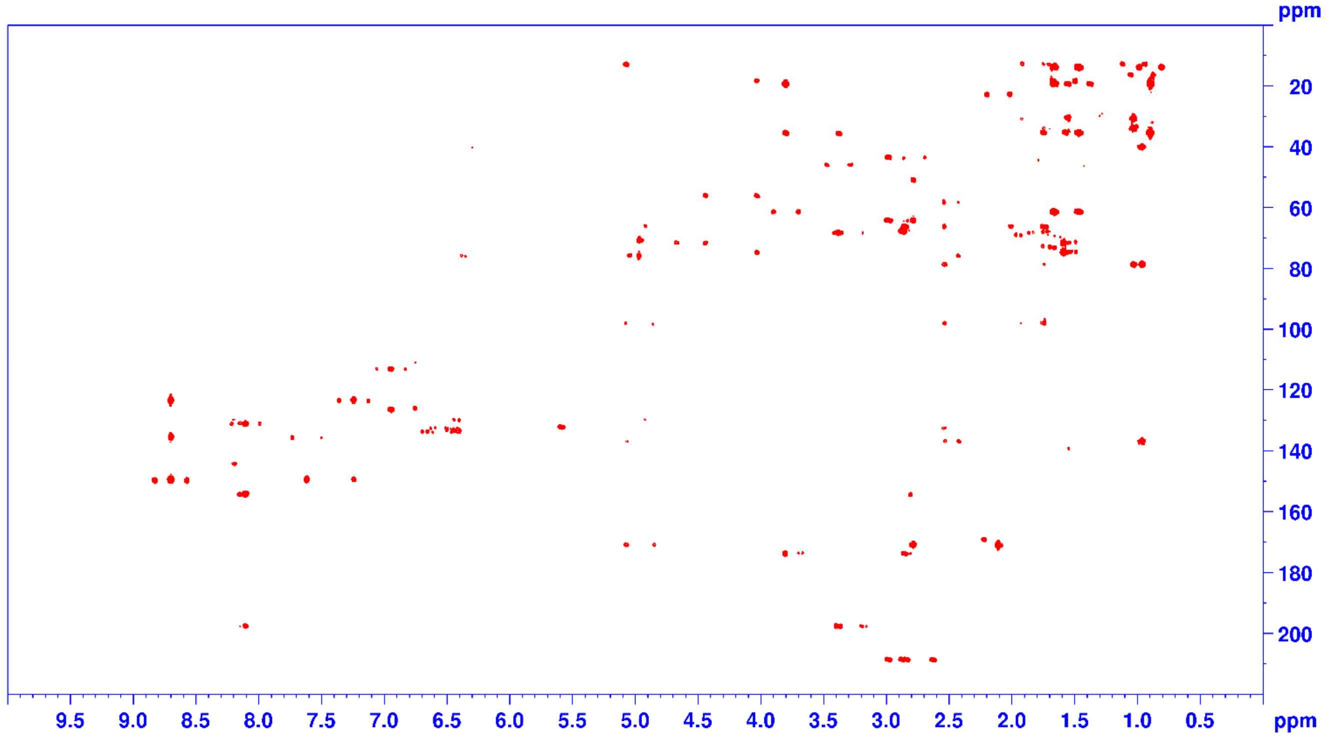

Figure S12. The ^{1}H-^{13}C HMBC spectrum of iso-partricin B.

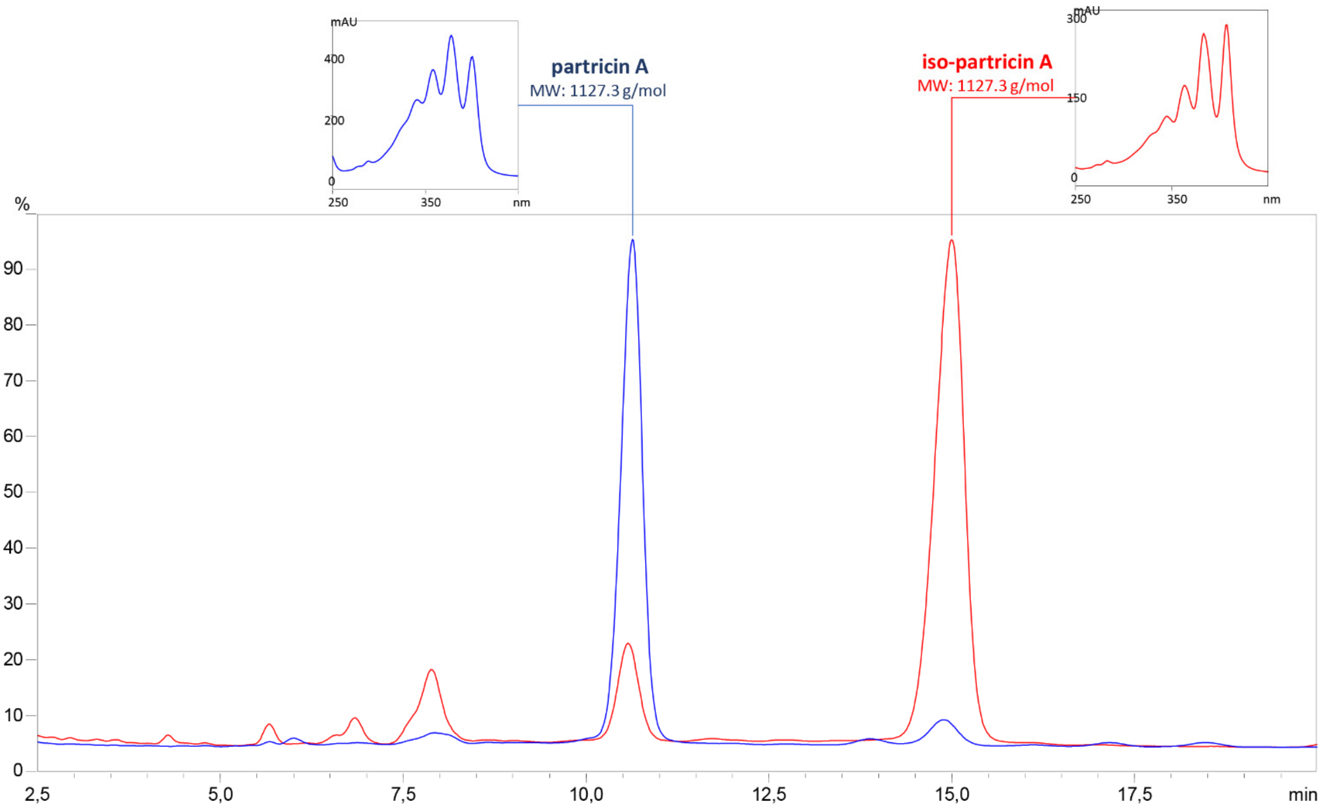

Figure S13. HPLC-DAD-ESIMS chromatogram of isolated iso-partricin A.

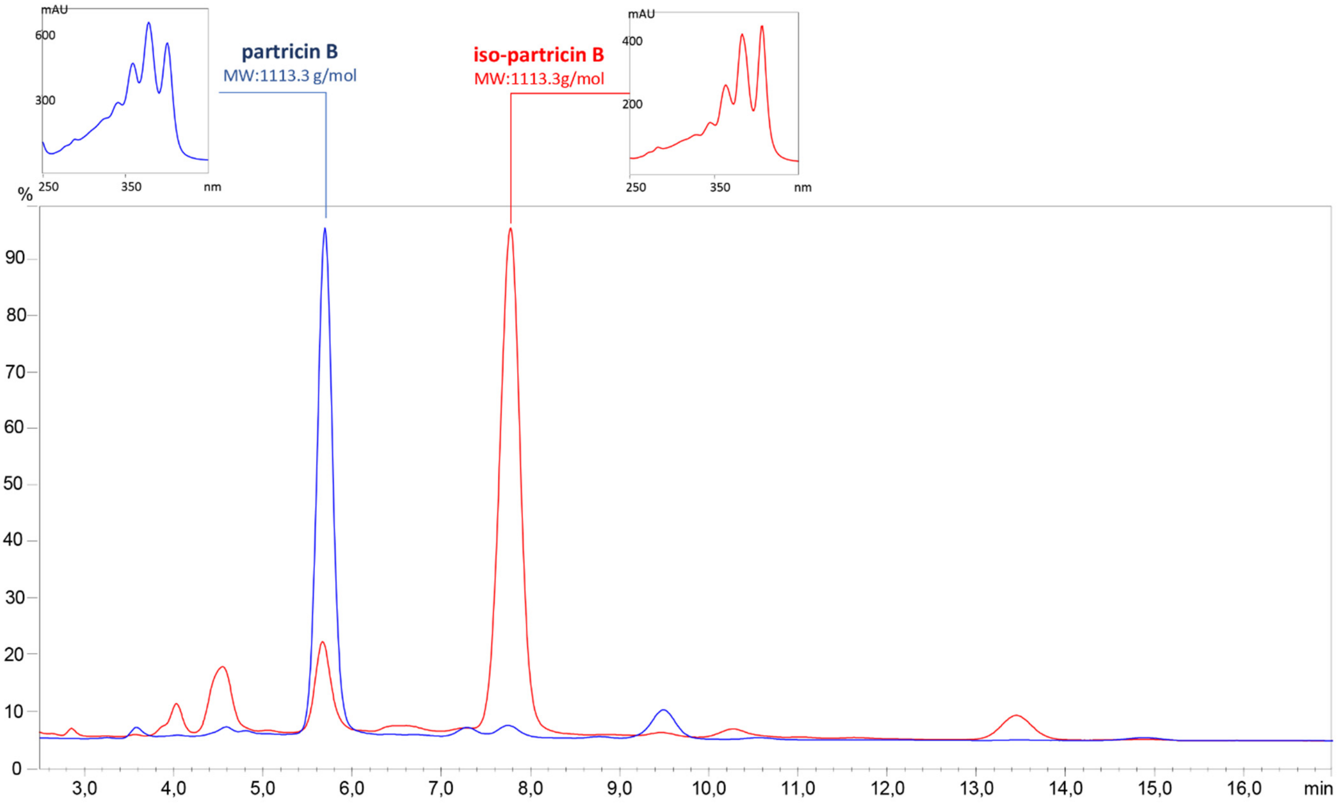

Figure S14. HPLC-DAD-ESIMS chromatogram of isolated iso-partricin B.
